# Measuring Mental Health in 2 Brazilian University Centers: Protocol for a Cohort Survey

**DOI:** 10.2196/63636

**Published:** 2025-03-14

**Authors:** Talita Di Santi, Ariana Gomes Nascimento, Pedro Fukuti, Vinnie Marchisio, Gian Carlo Araujo do Amaral, Camille Figueiredo Peternella Vaz, Luiz David Finotti Carrijo, Lilian Cristie de Oliveira, Luiz Octávio da Costa, Elisângela Mancini Marion Konieczniak, Luana Aparecida Zuppi Garcia, Vanessa Cristina Cabrelon Jusevicius, Eduardo de Castro Humes, Paulo Rossi Menezes, Euripedes Miguel, Arthur Caye

**Affiliations:** 1 Department of Psychiatry Faculty of Medicine University of São Paulo São Paulo Brazil; 2 National Center for Research and Innovation in Mental Health Sao Paulo Brazil; 3 Department of Pediatrics Faculty of Medicine University of São Paulo São Paulo Brazil; 4 Max Planck University Center Indaiatuba Brazil; 5 Jaguariúna University Center Jaguariuna Brazil; 6 Department of Psychiatry Faculty of medicine Federal University of Rio Grande do Sul Porto Alegre Brazil

**Keywords:** study design, university students, mental health screening, longitudinal survey, college students

## Abstract

**Background:**

Global concern for the mental well-being of university students is on the rise. Recent studies estimate that around 30% of students experience mental health disorders, and nearly 80% of these individuals do not receive adequate treatment. Brazil, home to around eight million university students, lacks sufficient research addressing their mental health. To address this gap, we aim to conduct a longitudinal mental health survey at 2 Brazilian universities.

**Objective:**

This paper outlines the research protocol for a web-based mental health survey designed to assess the well-being of Brazilian university students.

**Methods:**

The survey targets undergraduate students (N=8028) from 2 institutions: UniFAJ (Centro Universitário de Jaguariúna) and UniMAX (Centro Universitário Max Planck). Students will be invited to respond to self-reported questionnaires, including theSMILE-U (lifestyle and quality of life), the *DSM-5* (*Diagnostic and Statistical Manual of Mental Disorders* [Fifth Edition]) self-rated level 1 cross-cutting symptom measure, and a brief version of the Adult Self-Report Scale for attention-deficit/hyperactivity disorder. Students who exceed thresholds for conditions such as depression, anxiety, and attention-deficit/hyperactivity disorder will receive additional diagnostic instruments. The survey will be conducted annually, tracking individual and group trajectories and enrolling new cohorts each year. Data will be analyzed using cross-sectional and longitudinal methods, focusing on descriptive, associative, and trajectory analyses.

**Results:**

The first wave of data collection began in February 2024 and is expected to conclude in December 2024. As of October 2024, a total of 2034 of 7455 (27.2 in 100) eligible students had completed the questionnaire. Cross-sectional statistical analysis is planned to commence immediately after data collection and is expected to be completed by June 2025.

**Conclusions:**

This survey uses a scalable, cost-effective design to evaluate mental health conditions among Brazilian university students. The longitudinal framework facilitates the monitoring of mental health trends, supports the development of targeted interventions, and informs policy initiatives in higher education.

**Trial Registration:**

OSF Registries OSF.IO/AM5WS; https://doi.org/10.17605/OSF.IO/AM5WS

**International Registered Report Identifier (IRRID):**

DERR1-10.2196/63636

## Introduction

The mental health of university students is a widely recognized global concern. The transition to university life marks a crucial developmental phase characterized by individuation, the establishment of new social connections, and increased autonomy and responsibility [1]. This period aligns with continued, rapid brain development at a time when university students are exposed to multiple risk factors known to affect mental health, including psychosocial stressors, recreational drug use, alcohol binging, and disruptions in sleep patterns [2]. Mental health disorders typically present before or during young adulthood, often going unrecognized for years, resulting in significant delays in receiving treatment. Failing to adequately address mental health issues in a timely fashion can lead to the progression of more complex outcomes, such as school dropout, addiction, and self-harm. Indeed, the international prevalence estimates of mental health disorders reveal higher rates among college students compared to the general population. For example, a multicenter study involving 13,984 students from 8 countries, led by the World Health Organization (WHO), demonstrated that one-third met the clinical criteria for a psychiatric disorder in the previous year [3]. The most frequently reported were depressive disorders (18.5%) and anxiety disorders (16.7%-18.6%), followed by alcohol (6.8%) and other substance use disorders (3%). Moreover, approximately 22.6% of university students reported experiencing suicidal thoughts. Tragically, suicide stands as the second leading cause of death within this population [4].

The presence of psychiatric disorders is linked to numerous detrimental consequences both in the short and long term. In the short term, individuals may experience a decline in their quality of life, poorer academic performance, increased absenteeism, and a higher likelihood of course dropout [5-7]. In the long term, individuals endure lower quality of life, higher unemployment rates, and socioeconomic impairment [8,9].

Though the alarming data presented above were derived from studies conducted in countries with diverse income levels, to date, there is a disproportionately limited body of evidence addressing the mental health issues of young adults attending universities in low- and middle-income countries. The few studies that have been conducted in Brazil have tended to focus on medical students, also unveiling concerns about rates of mental distress in this population [10] with approximately 37% of them undergoing psychiatric treatment. In a study by Campos et al [11], the most prevalent diagnoses were depression (39.1%) and anxiety disorders or phobias (33.2%) and 4.5% declare previous suicide attempts. Severe mental health disorders such as psychotic disorders (3.7%) and bipolar disorder (1.9%) were less common [11].

In Brazil, there are approximately eight million students enrolled in 2714 higher education institutions [12]. Extrapolating from international prevalence estimates, over two million Brazilian university students may be struggling with mental health issues.

UniFAJ (Centro Universitário de Jaguariúna) and UniMAX (Centro Universitário Max Planck) are private university centers located in the medium-sized cities of Jaguariúna and Indaiatuba in the southeast of Brazil. Together, they serve approximately 8000 students enrolled in undergraduate programs spanning technical fields (eg, administration, accounting, architecture, law, or engineering) and health sciences (eg, medicine, nursing, psychology, biomedicine, or veterinary medicine). These institutions are representative of the broader Brazilian university population, as more than 75% of Brazilian students are enrolled in private universities.

To address the gaps in national and international literature, we propose to measure the mental health of all undergraduate students of UniFAJ and UniMAX. The survey will assess the prevalence of psychopathological symptoms and identify associated factors. The survey will also address another largely unexplored facet of mental health issues, namely the longitudinal course of mental health and quality of life. To achieve these goals, we designed a web-based mental health survey suitable for Brazilian students. This paper aims to describe the protocol and methods for conducting a web-based mental health cohort study in 2 private Brazilian universities. We hypothesize that, consistent with international studies, we will find high rates of common mental disorders, such as anxiety and depression. Furthermore, we expect to identify distinct patterns of mental health disorders unique to the low- and middle-income context of Brazil. By following students over time, we hypothesize that mental health disorders will correlate with poorer academic performance and quality of life, consistent with findings in the international literature.

## Methods

### Overall Design

This study will use validated self-report questionnaires delivered through an electronic web-based survey. The protocol will be repeated annually starting in 2024, allowing for the evaluation of mental health trajectories over time and the inclusion of new cohorts of incoming students to assess potential trends specific to these groups. This study of the university population is an arm of a large mental health project carried out by the National CISM (Center for Research and Innovation in Mental Health) [13]. CISM aims to study and expand, over the next 10 years, knowledge about mental health conditions in the State of São Paulo, the biggest one in Brazil.

### Participants and Recruitment

All undergraduate students enrolled at UniFAJ and UniMAX will be invited to participate annually. Invitations will be sent via email, providing a brief explanation of this study’s objectives and encouraging participation through the electronic survey link.

We have devised several strategies aimed at maximizing participation. First, we will conduct wide media campaigns in the university to promote awareness concerning mental health and discuss the importance of this study. Second, professors of all disciplines will be encouraged to remind students to engage in the survey. Third, nonresponders and survey noncompleters will receive at least 3 invitation reminders via email, followed by 3 reminders via text message (WhatsApp; Meta Platforms). Importantly, students will be informed that the time spent on the survey will count as an equivalent complementary academic activity. All these steps will be executed anonymously to ensure students’ privacy.

Upon receiving the invitation, students are required to read and sign an informed consent form (ICF). The consent form will clearly state that the participation is voluntary and that declining will not affect their academic standing or relationship with the university. Additionally, students who opt out will not receive further invitations.

The exclusion criteria are being younger than 18 years of age and limitations in accessing or responding to the survey (ie, no access to electronic devices or internet connection or being illiterate). These exclusions are expected to be negligible within this study population.

### Instruments

**Figure 1:**
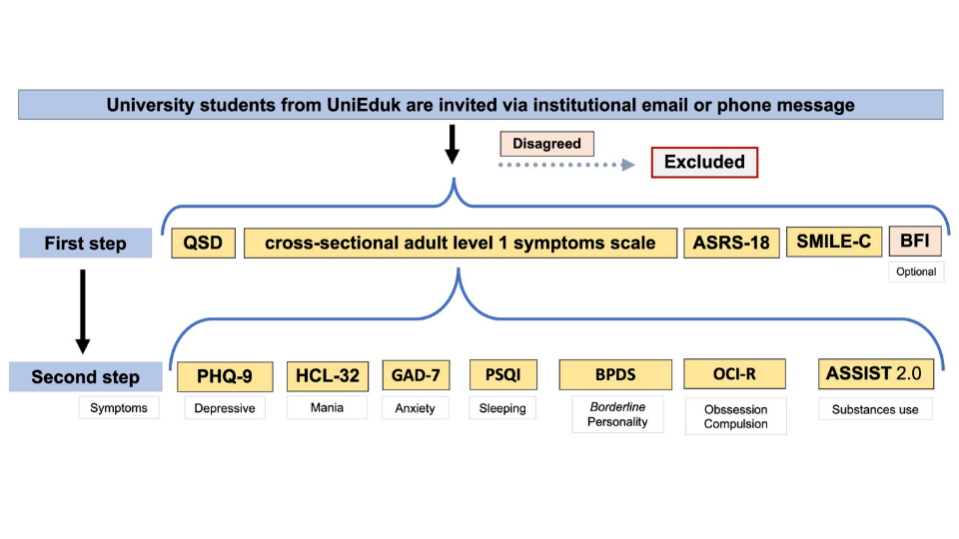
Initial data collection scheme: The figure illustrates the initial data collection scheme, and the scales applied according to the screening presented. ASSIST 2.0: Alcohol, Smoking and Substance Involvement Screening Test; ASRS-18: Adult Self Report Scale; BFI: Big Five Inventory; BPDS: Borderline Personality Disorder Scale; GAD-7: Generalized Anxiety Disorder Scale-7; HCL-32: Hypomania Checklist scale; OCI-R: Obsession and Compulsion Inventory; PHQ-9: Depression Patient Health Questionnaire-9; PSQI: Pittsburgh Sleep Questionnaire; QSD: Sociodemographic Questionnaire; SMILE-C: University Short Multidimensional Inventory Lifestyle Evaluation.

We selected empirically validated psychometric self-report questionnaires, all of which have been translated and validated for Brazilian Portuguese and can be administered electronically. A key challenge in designing this survey was balancing the need for a comprehensive assessment of the targeted phenotypes with the potential impact of a lengthy survey on participant engagement. To address this, we adopted a 2-step strategy aimed at minimizing survey duration while maintaining comprehensiveness (Figure 1). In the first step, in addition to collecting sociodemographic and overall lifestyle or quality of life information in Short Inventory Lifestyle Evaluation [14], we will screen for an array of mental health conditions using the *DSM-5* (*Diagnostic and Statistical Manual of Mental Disorders* [Fifth Edition]) cross-sectional adult symptoms scale level 1 [15] and a short version of the Adult Self Report Scale for attention-deficit/hyperactivity disorder [16] (Table 1). In the second step, participants scoring above predefined thresholds in any domain will be invited to complete domain-specific psychometric scales. These scales assess conditions such as depression, mania, generalized anxiety, sleeping disorders, borderline personality disorder, obsessive-compulsive disorder, attention-deficit/hyperactivity disorder, substance use (Multimedia Appendix 1). After completing the relevant scales, participants will have the option to answer a questionnaire on personality traits (the Big Five Inventory [17]). The total time required to complete the survey will vary depending on the domains assessed, ranging from approximately 20 to 40 minutes. This streamlined approach ensures a balance between thorough mental health assessment and participant engagement by limiting survey fatigue.

**Table 1:** Instruments used in step 1.

Instruments	Description
Sociodemographic Questionnaire (QSD)	The questionnaire was developed specifically for this study, including age, sexual identity and orientation, income, academic course, professional and career expectations, religiosity, use of social networks, and medical, psychiatric, or psychotherapeutic history.
The University Short Multidimensional Inventory Lifestyle Evaluation (SMILE-C)	Multidimensional assessment of lifestyle in 7 domains (diet, substance use, physical activity, stress management, social relationship, sleep, and screen time), along with an overall lifestyle score. The instrument comprises 24 questions that evaluate the frequency of behaviors considered healthy, with a response scale ranging from 0 to 4, where a higher score corresponds to a healthier lifestyle.
Adult Self Report Scale (ASRS-18)	Assesses symptoms of attention-deficit/hyperactivity disorder (ADHD) in adults, over the past 6 months, via 18 items divided into 2 domains: A (inattention) and B (hyperactivity-impulsivity). Responses for domain A vary as follows: 0=never, 1=rarely, 2=sometimes, 3=often, and 4=very often. As a screening measure, all participants will complete only the short version of this scale, which consists of 4 items (4, 5, 6, and 9) from part A and 2 items (1 and 5) from part B (hyperactivity). Those who score above 4 points on the short version will receive the complete version. Individuals are considered to have a possible diagnosis if they present at least 6 symptoms in at least 1 of the domains, or in both.
Cross-sectional adult level 1 symptoms scale (CSA) level 1 symptoms scale	Comprises 23 screening items that assess the frequency and intensity of symptoms across 13 domains of relevant symptomatology to frequent or severe psychiatric diagnoses. These domains include: sadness, irritability, mania, anxiety, somatic symptoms, suicidal ideation, psychosis, sleep disturbance, memory, repetitive thoughts and behaviors, dissociation, personality functioning, and substance use. Each item is rated on a 5-point scale (0=not at all; 1=very mild or rarely; 2=mild or several days; 3=moderate or more than half the days, and 4=severe or nearly every day).
Big Five Inventory (BFI)	The “Big Five” is an established model that analyzes 5 dimensions of personality: extroversion (tendency toward assertiveness and sociability), agreeableness (tendency toward reliability and altruism), conscientiousness (tendency to be careful and diligent), neuroticism (tendency toward negative emotions and sadness), and openness (tendency toward creativity and imagination). Likert-type scale with 44 items, where responses range from 1 (totally disagree), 2 (disagree a little), 3 (neither agree nor disagree), 4 (agree a little), to 5 (totally agree).

### Data Collection Instruments

Data collection will be performed using REDCap (Research Electronic Data Capture; Vanderbilt University), a secure digital platform designed for data management and research studies. REDCap facilitates the deployment of standardized digital instruments while ensuring participant anonymity [18]. The platform provides a convenient “survey queue” for participants to access the survey questionnaires and a “to-do list” so they can keep track of their progress. This allows for the tracking of initial participation, completeness status, and longitudinal data collection for all participants. The REDCap feature best suited to address automation of the communication process and is, furthermore, better at data collection is the automated invitations. The participants receive an individual link, and we can choose how many reminders will be sent as well as their periodization.

### Survey Distribution Tools

We will upload student data straight from a file containing the emails of all enrolled students (provided by the university’s administration sector), in order to generate a unique individual ID link for each student. This will allow us to track the participants’ survey engagement or completion rate.

### Reports and Alerts

**Figure 2:**
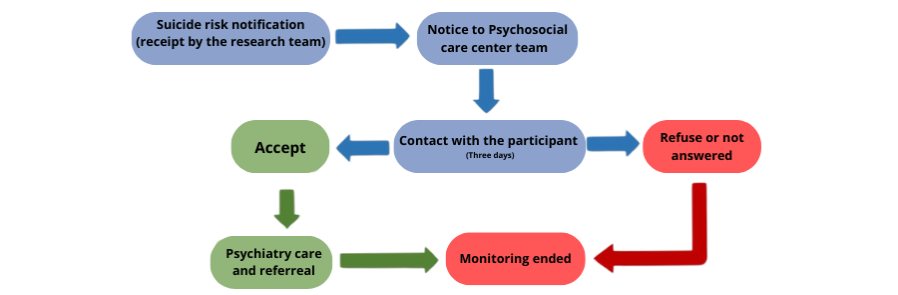
Suicide risk monitoring: The figure illustrates the monitoring protocol for participants identified as high risk of suicide.

The reports and alerts feature in REDCap will be used to monitor specific events and outcomes, particularly for suicide risk management (Figure 2). Suicide risk monitoring will rely on responses to the cross-sectional adult level 1 symptoms and PHQ-9 (Patient Health Questionnaire-9 depression) scales. The platform is programmed to generate immediate email alerts to this study’s team if participants affirmatively respond to either of 2 questions directly addressing suicidal ideation and suicide plans. This study’s team then immediately notifies a relevant health care team associated with our research group. Upon receiving this notification, the health care team will contact the participant to offer them appropriate psychiatric care. If the participant does not answer the first contact, the health care team will call again every day for 3 days. If the research participant accepts the offered care, they will be evaluated by psychiatry professionals and given necessary referrals. If they do not agree, this study’s team will register this nonagreement, and monitoring by the research team will end.

### Statistical Analyses

Given the annual assessments and longitudinal tracking of students throughout their university journey, we will use statistical methods suitable for both cross-sectional and longitudinal analyses. To enhance the representativeness of the sample, poststratification weights will be applied based on demographic data (eg, sex or age major) of the entire target sample provided by the university. All tests will be performed with a significance level of *P*=.05.

### Descriptive Analysis

We will perform descriptive analyses to characterize this study’s population. Summary statistics such as means, SDs, and medians will be computed for continuous variables (eg, age and quality of life) and frequencies and proportions for categorical variables (eg, gender or sexual orientation). Psychometric scales will be categorized into binary outcomes (absence or presence) based on established cutoff values.

### Simple Cross-Sectional Analyses

We will explore associations between sociodemographic characteristics, quality of life scores, personality traits, and the prevalence of psychopathological symptoms using parametric and nonparametric statistical tests (eg, chi-square tests for categorical variables, *t* tests [2-tailed], or Mann-Whitney *U* tests for continuous variables). For significant associations, odds ratios will be calculated to identify predictors of psychopathological symptoms. Linear or logistic regressions will be used to adjust for potential confounders, depending on the distribution of the outcome variable. Leveraging this study’s design and large sample size, we will use data to infer potential changes in mental health indicators throughout the students’ academic programs. This will involve comparing data from students at different stages of their academic journey (eg, first-year vs final-year students). To achieve this, generalized estimation equations will be used, with the independent variable year as a proxy for time. Models will control for confounding factors such as gender, age, and socioeconomic status.

### Longitudinal Analyses or Cohort Analyses

By tracking students longitudinally throughout their university journey, we aim to identify variations in mental health outcomes and analyze the evolution of symptomatology over time. Generalized estimation equations will be used, including time as an independent variable to model these changes and examine patterns at both individual and group levels.

### Ethical Considerations

This study adheres to the Code of Ethics of the World Medical Association (Declaration of Helsinki). This protocol was reviewed and approved by the Ethical Board Committee of the UniFAJ and UniMAX University Center (decision 6.153.870, 2023). This study follows the ethical principles outlined in Resolution 466/2012 of the National Health Council [19], which sets the guidelines and regulatory standards for research involving human beings in Brazil and complies with Law 14,874 which specifically governs human research ethics in the country. This study is registered in the Brazilian national system used to manage and oversee research involving human participants (Plataforma Brasil, under CAAE: 67251922.4.0000.0191), ensuring adherence to national regulations for research registration and monitoring.

Participation in this study is entirely voluntary. They will receive comprehensive information about the research objectives, methodology, and their rights. To ensure informed consent, this study uses a digital ICF via the REDCap e-consent platform. The ICF is presented in a clear and accessible format, explaining the purpose of the research and the voluntary nature of participation, and guarantees confidentiality and anonymity. Participants will be instructed to read the ICF and confirm their consent by responding to a specific question: “Have you understood the guidelines, and do you agree to participate freely, knowledgeably and spontaneously in this research?” If they agree, they are asked to enter their full name so that it can be attached to their acceptance to take part in this study. Additionally, the participant’s electronic signature will be collected, and a copy of the ICF will be provided via email. The participant will then be directed to a link to the digital survey. If they do not agree, the participant will receive a thank you note and the contact will be closed.

Participation in this study will be voluntary, with participants’ time and autonomy respected at all stages of the research. Participants may withdraw from this study at any time without any negative consequences or impact on their academic standing. This study involves completing a digital survey, ensuring a noninvasive and risk-free process. Study data will be deidentified to protect participants’ privacy and for data protection risks. No monetary compensation will be provided to the participants.

## Results

The first wave of data collection for this cohort began in February 2024 and is scheduled to conclude in December 2024. As of October 2024, a total of 2034 of 7455 (27.27%) eligible students had completed the questionnaire. Cross-sectional statistical analysis is planned to commence immediately after data collection and is expected to be completed by June 2025.

## Discussion

### Principal Findings

The psychological well-being of young adult college students is gaining significant attention, as it is one of the major determinants of their overall academic success, personal development, and prospects. A healthy lifestyle, access to mental health services, and the cultural relevance of mental health interventions are some of the themes that the university students consider relevant [20]. Meanwhile, the existing literature endures a dearth of evidence concerning the mental health of university students in low- and middle-income countries. We have presented the protocol of our cohort study, outlining the design of a web-based survey suited for Brazilian university students, aimed to improve our knowledge about the mental health of this population, mitigating an important literature gap.

In this way, we can address the hypotheses of this research. The prevalence of common mental disorders in the world is, as shown, high. A replication study for other universities in the country allows us to learn about the prevalence of these disorders and the risk factors in Brazil. The prior work of the WHO evaluated sociodemographic correlates of mental disorders among first-year university students. They also used web-based self-report questionnaires about *DSM-IV* (*Diagnostic and Statistical Manual of Mental Disorders* [Fourth Edition]) mental disorders: major depression, mania or hypomania, generalized anxiety disorder, panic disorder, alcohol use disorder, and substance use disorder. Their results show high rates, one-third of the students screened positive for 1 common mental disorder [3]. We had some similarities and differences with the study by Auerbach et al [3]. Our protocol is also a web-based self-report questionnaire that contains sociodemographic aspects and mental health scales. We access more sociodemographic aspects than the WHO study and, in the same way, we access more mental health disorders instead (Figure 1) of only common mental disorders. Finally, our research had been projected to follow these universities’ students, identifying some risk factors and consequences of mental health disorders.

The studies conducted in Brazil are mostly cross-sectional and their focus is on medical students [10]. The study by Miguel et al [10] showed higher rates of common mental disorders when in comparison to the WHO study [3]. Our study protocol describes a proposal to systematically and longitudinally survey the mental health of a large population of university students in Brazil. Our plan was based on methodological decisions that balance quality, precision, scalability, cost, and the likelihood of participant engagement.

Among the challenges encountered during the design of this web-based mental health survey, we have highlighted the need to assess a large number of potential participants and assess most psychopathological symptoms while aiming to be concise to achieve a maximum response rate and increase generalizability. Although university students are the majority in the digital world, they usually do not seek support [21]; therefore, another challenge is to engage them in web-based mental health surveys, through the many incentives we have described, we have accomplished a response rate of 2034 of 7455 (27.27%), which is relatively high compared to other web-based surveys.

Ethical considerations addressed here are related to the importance of interaction with the local health care services to provide assistance if cases of psychiatric emergency (ie, risk of suicide) are detected, as well as ensuring data privacy. We have also strived for this longitudinal study proposal to be as cost-efficient as possible considering financial constraints.

Our proposed use of an internet portal improved participant engagement and data integration and reduced longitudinal data collection’s time and expense [22,23]. Indeed, automated data capture minimizes the need for paid researchers to run participants and enter data, while also reducing data entry errors [24]; as our survey involves multiple questionnaires, the platform allows for easier and more engaging access for potential participants [25]. Our dissemination plan has internal importance for promoting and preventing mental disorders at UniFAJ and UniMAX universities. The results of the research can be shared with the students themselves and with the university coordinators, guaranteeing confidentiality. Another of our dissemination plans is to share this data and results with the media and with the governments.

Finally, we believe that this protocol could be useful for monitoring mental health cross-sectionally and longitudinally in thousands of universities across Brazil, either to assess their mental health in a cost-efficient manner or guiding interventions such as preventive mental health programs or even screening students with high risk of having mental disorder. This would allow a positive impact on the burden of mental health in university students and ultimately in our communities.

### Limitations

There are disadvantages associated with digital research. For example, open-ended questions cannot be explored with immediate follow-up questions and participants are unable to seek clarification of ambiguous items [26]. To address this issue, in the present survey, participants can easily email the project team with any questions they may have.

Selection bias presents another challenge for digital research. Internet access is affected by myriad variables including income, geographical location, mental health status, and age [27,28]. We do not anticipate serious issues with internet accessibility. UniFAJ and UniMAX have free internet access for all students. In addition, there were 181.8 million internet users in Brazil at the beginning of 2023, which means it is 84.3% of the population [29]; and the southeast, where UniFAJ and UniMAX are, has an even greater user concentration. Our plan to remind participants via WhatsApp to finish their questions is sound, as WhatsApp is the most used social media by Brazilians, around 169 million [29]. Thus, though we cannot fully account for all these possibilities, the present strategy maximizes accessibility in a way that will mitigate these potential confounding issues and, consequently, increase the response rate.

After identifying participants, the present protocol contains strategies to maximize the probability that participants will continue to engage with and complete the survey. For example, one way to increase engagement is to provide participants with information about this study that (1) knows their interests, (2) helps them understand the importance of their participation in mental health research, and (3) increases their confidence about participating. To this end, the research team will hold meetings with UniFAJ and UniMAX course coordinators to transmit detailed study information so that it reaches the students. Furthermore, a 2-minute video will be sent to all participants. This video contains information about the research team, the reason for the research and its objectives, details about the questionnaires (application time, confidentiality, or freedom to decline), and the opportunity to ask questions via email. We intend to demonstrate the importance of participation in promoting mental health among the university population.

The security of collected data is ensured by the REDCap platform, a trusted and secure data collection and storage platform [30] used throughout the scientific community. The platform allows for the long-term reduction of research costs, the possibility of use on many devices, and rapid data entry, review, and analysis [31].

As noted in the Introduction section, there is a paucity of data that have been collected from low- and middle-income countries. Existing studies were carried out mainly with students from specific courses [32]. The data analysis will summarize the data collected and integrate information about the mental health of this population. It will allow us to better understand the mental health of the Brazilian university population This survey is a structured means for assessing this population’s mental health given that it has been well established that preventive actions are feasible, cost-effective, and efficient in improving overall mental health [33] and this period of life represents a hub that critically impacts their responsibilities, mental health, values, and outcomes [34]. We believe that the results of this survey could in the future guide policy makers in the design and implementation of preventive programs destined specifically for this public group and ultimately have a positive impact on the mental health of our communities.

### Conclusion

College and university students have high rates of mental health issues. We have developed and described a web-based mental health survey that will allow us to evaluate and detect these issues with low cost and reasonable response rate in a university in Brazil. These efforts will allow us, soon, to monitor and test the efficiency and impact of mental health preventive programs. The accurate and representative data about mental health disorders, their risk factors, and the quality of life of these universities’ students can lead the path for new policies to ensure mental health and quality of life for these populations.

This model could be scaled up across other universities in Brazil to easily assess the mental health status of their students and have a significant impact on the mental health of our communities.

## Appendix

Multimedia Appendix 1 Instruments to be used in step 2.

## Abbreviations

CISM: Center for Research and Innovation in Mental Health
DSM-5: Diagnostic and Statistical Manual of Mental Disorders [Fifth Edition]
DSM-IV: Diagnostic and Statistical Manual of Mental Disorders [Fourth Edition]
ICF: informed consent form
PHQ-9: Patient Health Questionnaire-9
REDCap: Research Electronic Data Capture
UniFAJ: Centro Universitário de Jaguariúna
UniMAX: Centro Universitário Max Planck
WHO: World Health Organization

## Data Availability

Data sharing is not applicable to this article as no data sets were generated or analyzed during this study.
