# Supplementary material for: Measuring Mental Health in 2 Brazilian University Centers: Protocol for a Cohort Survey
Source: JMIR Res Protoc. 2025 Mar 14;14:e63636. doi: 10.2196/63636 (PMC11953593; doi:10.2196/63636)
Supplement: Multimedia Appendix 1 [file resprot_v14i1e63636_app1.docx]

**Table S1**. Instruments to be used in step 2.

| Scale | Screening | Description |
| --- | --- | --- |
| Depression Patient Health Questionnaire-9 (PHQ-9) | Mild or higher score in domain 1 | Consists of 9 items corresponding to the diagnostic criteria for depression disorder from the DSM-IV-TR^a^. Symptoms are assessed based on the previous 2 weeks and the intensity is measured on a Likert scale of 0 to 3 points, corresponding to responses of 0=not at all, 1=several days, 2=more than half the days, and 3=nearly every day. The tenth question evaluates the impact of these symptoms on daily activities, such as work and study. A score equal to or above 10 indicates a possible depressive episode. |
| Hypomania Checklist scale (HCL-32) | Mild or higher score in domain 3 | Designed for screening manic symptoms during periods when participants have experienced excitement, euphoria, or elevated mood. The questionnaire consists of 2 multiple-choice items and 32 dichotomous response items (yes or no). Each “yes” response is equivalent to 1 point, and total scores exceeding 18 indicate possible bipolar disorder. |
| Generalized Anxiety Disorder Scale-7 (GAD-7) | Mild or higher score in domain 4 | Consists of 7 items corresponding to the diagnostic criteria for Generalized Anxiety Disorder from the DSM-IV-TR. The items assess how much the participant has been bothered by those symptoms in the past 2 weeks. The intensity is measured on a Likert scale of 0 to 3 points, corresponding to responses of 0=not at all, 1=several days, 2=more than half the days, and 3=nearly every day. Scores between 10-14 correspond to moderate symptoms, and scores above 15 indicate severe symptoms. |
| Pittsburgh Sleep Questionnaire (PSQI) | Mild or higher score in domain 8 | Assesses the quality of sleep over the past month and provides an index of the severity and nature of sleep problems. The instrument consists of 19 self-administered questions grouped into 7 components. It is a Likert-type scale ranging from 0 to 3, corresponding to 0=never, 1=less than once a week, 2=once or twice a week, and 3=3 or more times a week. A score equal to or greater than 5 is indicative of sleep problems. |
| Borderline Personality Disorder Scale (BPDS) | Very mild or greater score of suicidal ideation (domain 6), or mild or greater score of somatic symptoms (domain 5) dissociation (domain 11), personality functioning (domain 12) | Instrument for the evaluation of borderline disorder typical symptoms. It consists of 23 items where individuals reflect on symptoms experienced in the week before the assessment. This scale uses a Likert-type scale ranging from 0 to 4, with 0=not at all, 1=a little, 2=considerably, 3=very, and 4=very strongly. Scores above 16 suggest significant symptoms. |
| Obsessive thoughts and behavior Obsession and Compulsion Inventory (OCI-R) | Mild or higher score in domain 10 | Self-administered scale for assessing obsessive-compulsive symptoms. It comprises 18 items that assess the intensity of symptoms using a Likert-type scale ranging from 0 to 4, where 0=not at all, 1=a little, 2=moderately, 3=very, and 4=extremely. Scores above 18 indicate the possibility of obsessive-compulsive disorder. |
| Alcohol, Smoking and Substance Involvement Screening Test (ASSIST 2.0) | Very mild or higher score in domain 13 | Consists of 8 questions about the use of 9 classes of psychoactive substances (tobacco, alcohol, marijuana, cocaine, stimulants, sedatives, inhalants, hallucinogens, and opioids). The questions cover the lifetime frequency of use as well as the prior 3 months, problems related to use; concern about the use by people close to the user, impairment in the performance of expected tasks, unsuccessful attempts to quit or reduce use, feelings of compulsion, and injectable use. Each response corresponds to a score ranging from 0 to 4, with a total sum ranging from 0 to 20. Scores in the range of 0 to 3 are considered indicative of occasional use, scores from 4 to 15 suggest abuse, and scores of ≥16 are suggestive of dependence. |

^a^DSM-IV-TR: Diagnostic and Statistical Manual of Mental Disorders, Fourth Edition—Text Revised.
